# A new basal hadrosaurid (Dinosauria: Ornithischia) from the latest Cretaceous Kita-ama Formation in Japan implies the origin of hadrosaurids

**DOI:** 10.1038/s41598-021-87719-5

**Published:** 2021-04-27

**Authors:** Yoshitsugu Kobayashi, Ryuji Takasaki, Katsuhiro Kubota, Anthony R. Fiorillo

**Affiliations:** 1 Hokkaido University Museum, Hokkaido University, Sapporo, Hokkaido 060-0810 Japan; 2 Faculty of Biosphere-Geosphere Science, Okayama University of Science, Okayama, 700-0005 Japan; 3 Museum of Nature and Human Activities, Hyogo, Sanda, Hyogo 669-1546 Japan; 4 Institute of Natural and Environmental Sciences, University of Hyogo, Sanda, Hyogo 669-1546 Japan; 5 Institute for the Study of Earth and Man, Southern Methodist University, Dallas, TX 75275 USA

**Keywords:** Evolution, Palaeontology, Phylogenetics, Speciation, Taxonomy

## Abstract

Here we describe a partial hadrosaurid skeleton from the marine Maastrichtian Kita-ama Formation in Japan as a new taxon, *Yamatosaurus izanagii* gen. et sp. nov., based on unique characters in the dentition. Our phylogenetic analysis demonstrates *Yamatosaurus izanagii* belongs to Hadrosauridae, composed of *Hadrosaurus foulkii* + (*Yamatosaurus izanagii* + (Saurolophinae + Lambeosaurinae)). The coracoid lacks a biceps tubercle as in non-hadrosaurid hadrosauroids, suggesting its presence is a key feature for the clade of Saurolophinae and Lambeosaurinae. The evolutionary rates analysis further supports that shoulder and forelimb features, which are likely to have been involved in locomotion, were important for the early evolution of Hadrosauridae. Our biogeographic analyses show that basal hadrosaurids were widely distributed in Asia and Appalachia, that the clade of Saurolophinae and Lambeosaurinae originated in Asia, and that eastern Asia may have served as a refugium of relict hadrosauroid taxa such as *Plesiohadros djadokhtaensis*, *Tanius sinensis,* and *Yamatosaurus izanagii* during the Late Cretaceous. The contemporaneous occurrence of basal (*Yamatosaurus izanagii*) and derived (*Kamuysaurus japonicus*) hadrosaurids during the Maastrichtian in Japan is the first record in Asia. Because of the long geographical distance between these localities, they likely did not co-exist, but instead demonstrate some level of provinciality.

## Introduction

Hadrosauroids were successful herbivorous ornithischian dinosaurs during the Cretaceous. The fossil record of known basal hadrosauroids is mainly from the Lower Cretaceous deposits of Europe and eastern Asia, whereas derived forms are known from the Upper Cretaceous rocks of all continents except Australia and the Indian subcontinent. A large clade of derived hadrosauroids, flourished in the post-Campanian, referred to in previous studies as the Euhadrosauria^1–3^, Hadrosauridae^4,5^, and Saurolophidae^6^, and consisting of two major clades with solid-crested (Hadrosaurinae^7^ or Saurolophinae^6^) and hollow-crested (Lambeosaurinae) skulls^2,6^. Hadrosauridae is defined as the most recent common ancestor of *Edmontosaurus regalis*, *Saurolophus osborni*, *Lambeosaurus lambei*, and *Hadrosaurus foulkii* and all of its descendants^6^. Hadrosaurids were successful in both abundance^8,9^ and cosmopolitan distribution that included the ancient Arctic^10–12^. The high diversification of hadrosaurids is attributed to their efficient oral processing system, established by specialized dentitions with complex grinding surface^13^ together with cranial kinetics^14,15^. Social behaviors, expressed by their unique supracranial crests, are also likely to have contributed to the success of the derived hadrosauroids^16–18^. In addition to their functional adaptations, Kobayashi et al.^19^ recently proposed that occupation of marine-influenced environments by ancestral hadrosaurids also played an important role in their early evolution. However, because the sister clades of Hadrosauridae have varied in previous studies (e.g., *Telmatosaurus transsylvanicus*, *Plesiohadros djadokhtaensis*, *Eotrachodon orientalis*, and *Lophorhothon atopus*), the rise of hadrosaurids has been obscure.

**Figure 1:**
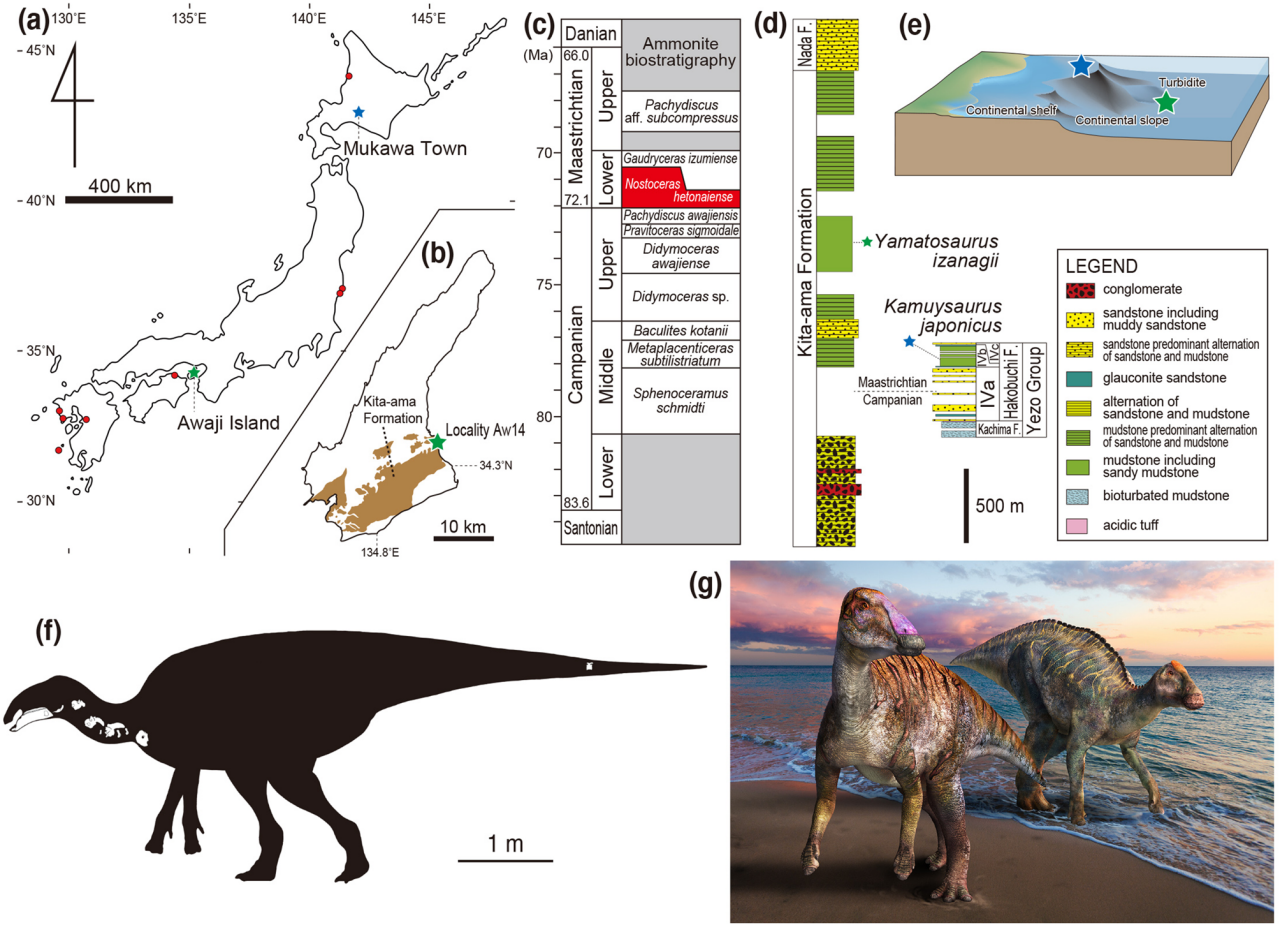
Map of Japan, showing the localities of *Yamatosaurus izanagii* gen. et sp. nov. on Awaji Island (green star), *Kamuysaurus japonicus* in Mukawa Town (blue star), and other Late Cretaceous hadrosauroids (red circles) (**a**) and the location of Locality Aw14 on Awaji Island (**b**). Ammonite biostratigraphy, showing the position of the *Nostoceras hetonaiense* Zone (**c**). Stratigraphic sections of the Kita-ama and Hakobuchi formations (**d**) and depositional environments of *Yamatosaurus izanagii* (green star) and *Kamuysaurus japonicus* (blue star) (**e**). Note that (**d**) differs from Fig. 1 of Tanaka et al.^27^ because we corrected errors, including the scale and the stratigraphic boundaries between the Kita-ama and Noda formations and between the Campanian and Maastrichtian. Silhouette of *Yamatosaurus izanagii*, showing recovered skeletal elements (**f**) (Courtesy of Genya Masukawa). Life reconstruction of *Yamatosaurus izanagii* (left) and *Kamuysaurus japonicus* (right) (**g**) (Courtesy of Masato Hattori). (**a**) and (**b**) were created by one of the authors of this paper, Katsuhiro Kubota, by using Adobe Illustrator 2021 (https://www.adobe.com/products/illustrator.html).

Among hadrosauroid remains from the Late Cretaceous in Japan, the best-preserved specimen is *Kamuysaurus japonicus* from the Maastrichtian Hakobuchi Formation, Yezo Group in Hokkaido Prefecture. Although other specimens are fragmentary and isolated elements such as teeth, limb elements, and vertebrae, it is noteworthy that hadrosauroid remains have been discovered from all four major islands (Hokkaido, Honshu, Shikoku, and Kyushu) of Japan^20^. In addition to these specimens, an amateur fossil collector, Mr. Shingo Kishimoto, discovered a partial hadrosauroid specimen in May 2004. This new discovery includes a dentary, a surangular, isolated dentary teeth, cervical vertebrae, a caudal vertebra, cervical ribs, and a coracoid (MNHAH D1-033516). This specimen was recovered from the Maastrichtian horizon of the Kita-ama Formation, Izumi Group, in Sumoto City of the Awaji Island in Hyogo Prefecture (Fig. 1a,b). MNHAH D1-033516 was initially identified as a lambeosaurine hadrosaurid^21^, but further study was recommended^19^. Here we provide the first description of MNHAH D1-033516 and test its taxonomic status. The new hadrosaurid provides insight into how coastal habitats likely impacted the early divergence of the Hadrosauridae and the coexistence of the Far East coastal hadrosauroids during the early Maastrichtian.

### Institutional abbreviations

AMNH (FARB): American Museum of Natural History (Fossil Amphibians, Reptiles, and Birds), New York, USA; CMN: Canadian Museum of Nature, Ottawa, Ontario, Canada; MNHAH: Museum of Nature and Human Activities, Hyogo, Sanda, Hyogo, Japan; MPC: Mongolian Paleontological Center, Ulaanbaatar, Mongolia; MOR: Museum of the Rockies, Montana, USA; NMMNH: New Mexico Museum of Natural History, Albuquerque, New Mexico, USA; ROM: Royal Ontario Museum, Toronto, Ontario, Canada.

## Results

### Geological setting

The new specimen was recovered from blocks of dark grey mudstones of the upper part of the Kita-ama Formation of the Izumi Group, exposed at a quarry (Locality Aw14, Morozumi^22^) in Yura Town, Sumoto City of the Awaji Island in Hyogo Prefecture. The upper part of the Kita-ama Formation, comprises inter-arc basin deposits including conglomerate, sandstone, and mudstone representing distal turbidite faces (Fig. 1c–e). The unit is rich in invertebrate fossils; the ammonoid *Nostoceras hetonaiense*, the nautilid *Eutrephoceras* sp., bivalves (e.g., *Apiotrigonia (Microtrigonia) amanoi*, *Eriphyla japonica*, and *Inoceramus* (*Endocostea*) *shikotanensis*), and gastropods (e.g., *Gigantocapulus problematicus*), decapods (e.g., *Archaeopus ezoensis*, *Hinecaris simplex*, and *Ahazianassa masanorii*)^22–24^. Fragmentary materials of vertebrates such as fishes, a turtle (*Mesodermochelys undulatus*), a mosasaur, and a hesperornithiform^23,25–27^ (Tanaka et al.^27^ erroneously listed pterosaur and plesiosaur materials from the locality) also have been reported from this quarry. Plant fossils from the quarry include angiosperms (*Platanus* sp. and *Ulmus* sp.) and cycad (*Zamiophyllum* sp.)^23^. The exposed beds at the quarry are within subchron 32.1r^28^, which is currently dated as 71.94–71.69 Ma (early Maastrichtian)^29^, as well as the *Nostoceras hetonaiense* Zone^22^, which is the same as the ammonite zone that includes *Kamuysaurus japonicus*. This ammonite zone is currently dated as 72.4–70.6 Ma (based on the dates of the underlying *Didymoceras awajiense* Zone^30^ and overlying *Gaudryceras izumiense* Zone^31^), which is in concordance with the magnetostratigraphic data.

### Systematic paleontology

- Dinosauria Owen^32^.
- Ornithischia Seeley^33^.
- Ornithopoda Marsh^34^.
- Hadrosauridae Cope^35^.
- *Yamatosaurus izanagii* gen. et sp. nov.
- urn:lsid:zoobank.org:act:1C728683-C3EA-4688-9420-096D13EB6227
- urn:lsid:zoobank.org:act:15719C4B-D00E-4907-B82A-9661799498A3

### Nomenclatural Acts

This published work and the nomenclatural acts it contains have been registered in ZooBank, the proposed online registration system for the International Code of Zoological Nomenclature. The ZooBank life science identifiers (LSID) can be resolved and the associated information viewed by appending the LSID to the prefix http://zoobank.org/. The LSID for this publication is: urn:lsid:zoobank.org:pub:ECCF9E80-C6CC-4D14-966A-F9B79B2395B9.

### Etymology

“*Yamato*” refers to the ancient name for a region of the Japanese archipelago, including the western half of the main island (Honshu Island), Shikoku Island, and the northern half of Kyushu Island, ruled by the Yamato Kingdom from the third to the seventh century. “*Sauros*” means reptiles. The specific name, “*izanagi*”, refers to a deity in Japanese mythology, which created eight countries of Yamato with another deity, Izanami, based on the oldest history book in Japan called “Kojiki (Records of Ancient Matters)”, published in 712 CE (Common Era). The first country created was the Awaji Island, followed by the Shikoku, Oki, Kyushu, Iki, Tsushima, Sado, and Honshu islands.

### Holotype

MNHAH D1-033516, a right dentary, a right surangular, twelve isolated dentary teeth, four cervical vertebrae, a distal caudal vertebra, three cervical ribs, and a coracoid. This specimen is stored in the Museum of Nature and Human Activities, Hyogo, Sanda City, Hyogo Prefecture, Japan.

### Locality and horizon

Locality Aw14 (Morozumi^22^) of Yura Town, Sumoto City of the Awaji Island, Hyogo Prefecture, Japan; the early Maastrichtian (71.94–71.69 Ma)^29^ Kita-ama Formation of the Izumi Group.

### Diagnosis

A hadrosaurid with unique characters in having only a single tooth as a minimum number of functional teeth per tooth position in the middle of the dentary dental battery (9th, 11th, 14th, 16th, 19th, 21st, and 23rd tooth positions) and the complete absence of the “branched ridges” on the dentary tooth occlusal surfaces. It is also unique in the combination of the additional following characters: the low angle between the dentary symphysis and lateral surface of the dentary and ventrally facing ventral surface of the surangular.

### Description

**Figure 2:**
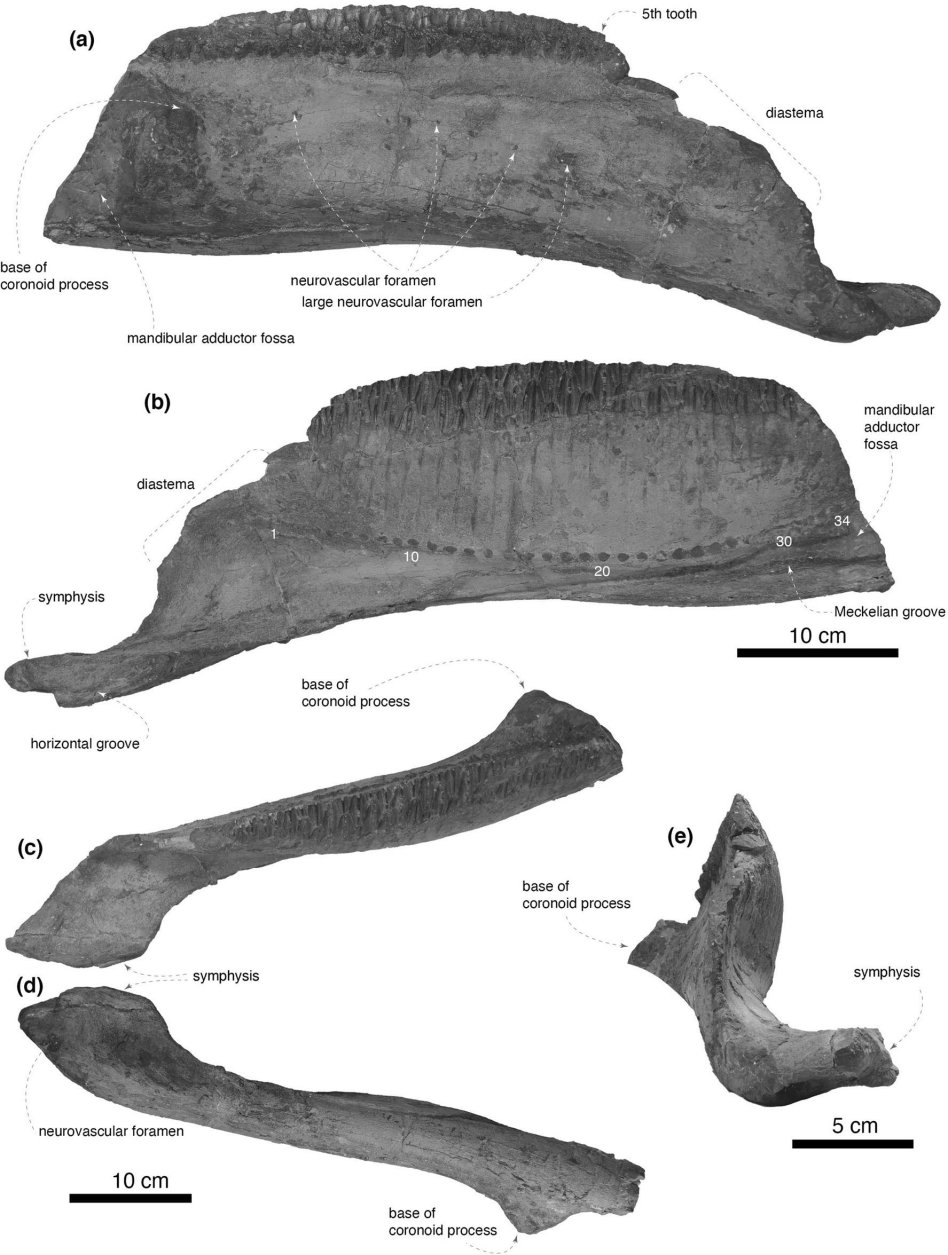
Right dentary of *Yamatosaurus izanagii* gen. et sp. nov. in lateral (**a**), medial (**b**), dorsal (**c**), ventral (**d**), and anterior (**e**) views. Numbers in white in (**b**) indicate the positions of nutrient foramina.

The right dentary is nearly complete, missing its posterior end and the coronoid process. The lateral surface of the main body of the dentary bears multiple neurovascular foramina. A large foramen is positioned at the level of the seventh dentary tooth, and several smaller foramina are present posteriorly. In lateral view, the anterior portion of the dentary is downturned, and an angle between the ventral edge of the anterior portion and a horizontal plane, or tooth row, is 20 degrees (Fig. 2a). In medial view (Fig. 2b), the long axis of the tooth row is parallel to the ventral margin of the middle region of the dentary main body as in all hadrosaurids except for the Brachylophosaurini^36^. A thin alveolar parapet, covering the medial surface of the dental battery, is dorsoventrally taller posteriorly than anteriorly. The nutrient foramina are organized in a shallow arch ventral to the dental battery. The Meckelian groove extends along the ventral margin of the posterior two-thirds of the dentary. The ventral edge of the dentary main body below the coronoid process is weakly bowed. In dorsal view, the lateral margin of the occlusal surface of the dental battery is straight and parallel to the lateral surface of the dentary main body (Fig. 2c).

The dentary diastema (edentulous region of the dentary, defined by Horner^37^) is approximately 99 mm in anteroposterior length. This is 30% of the length between the anterior end of the dental battery and the posterior margin of the coronoid process (330 mm). Although the coronoid process is not preserved, the length was estimated from the base of the coronoid process. The dorsal margin of the dentary, where the lateral ramus of the predentary contacts, is strongly concave in lateral view and is angled by 110° from a horizontal plane (Fig. 2b).

The symphysial process of the dentary curves medially at nearly a right angle (Fig. 2e) as in non-hadrosaurid hadrosauroids (e.g., *Plesiohadros djadokhtaensis*; MPC-D 100/745) and some members of Lambeosaurini, including *Olorotitan arharensis*^38^ and *Parasaurolophus tubicen* (NMMNH P-25100). The maximum mediolateral width of the dentary symphysial region in dorsal view is 81 mm, which is approximately twice as wide as the minimum breadth of the dentary posterior to the dentary symphysial region (Fig. 2c). In dorsal view, the symphysis is largely divergent from the lateral surface of the main body of the dentary by an angle of 20 degrees as in *Eotrachodon orientalis*^39^ but unlike a nearly parallel alignment in hadrosaurids such as *Edmontosaurus regalis*^40^ and *Parasaurolophus tubicen* (NMMNH P-25100). The symphysis bears a shallow horizontal groove for contact with its counterpart (Fig. 2b). The ventral surface of the symphysial process has a large neurovascular foramen (Fig. 2d).

The base of the coronoid process is laterally expanded as in hadrosaurids and some non-hadrosaurid hadrosauroids such as *Plesiohadros djadokhtaensis*, *Nanningosaurus dashiensis*, and *Adynomosaurus arcanus*^41–43^ and is separated from the dental battery (Fig. 2e). The posteromedial surface of the coronoid process is largely excavated, forming the mandibular adductor fossa.

**Figure 3:**
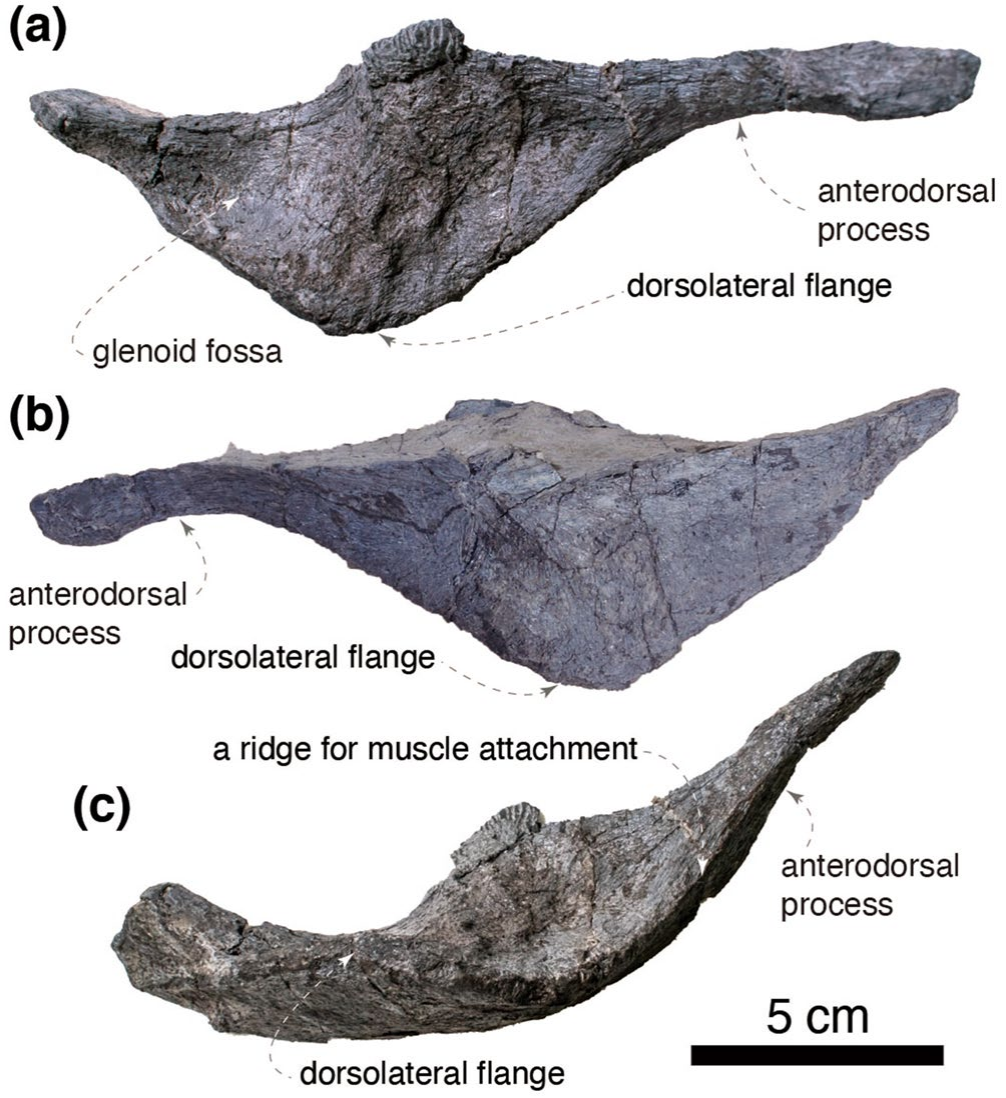
Right surangular of *Yamatosaurus izanagii* gen. et sp. nov. in dorsal (**a**), ventral (**b**), and lateral (**c**) views.

The dorsolateral portion of the right surangular is preserved (Fig. 3). The preserved region of the anterodorsal process is thin and bears a well-defined ridge for the attachment of *m. adductor mandibulae externus superficialis*^44^. The ridge is posteriorly continuous with the dorsolateral flange, which is gently arcuate in dorsal view. The lateroventral surface of the surangular bears no foramen as in derived hadrosauroids including *Bactrosaurus johnsoni*^45,46^. The dorsal surface of the anterior half of the surangular is concave to form the mandibular adductor fossa, whereas the posterior half forms a glenoid fossa that receives the mandibular condyles of the quadrate. The ventromedial portion of the surangular is lost before collection thus the morphology of the caudal process and the retroarticular process is unavailable.

**Figure 4:**
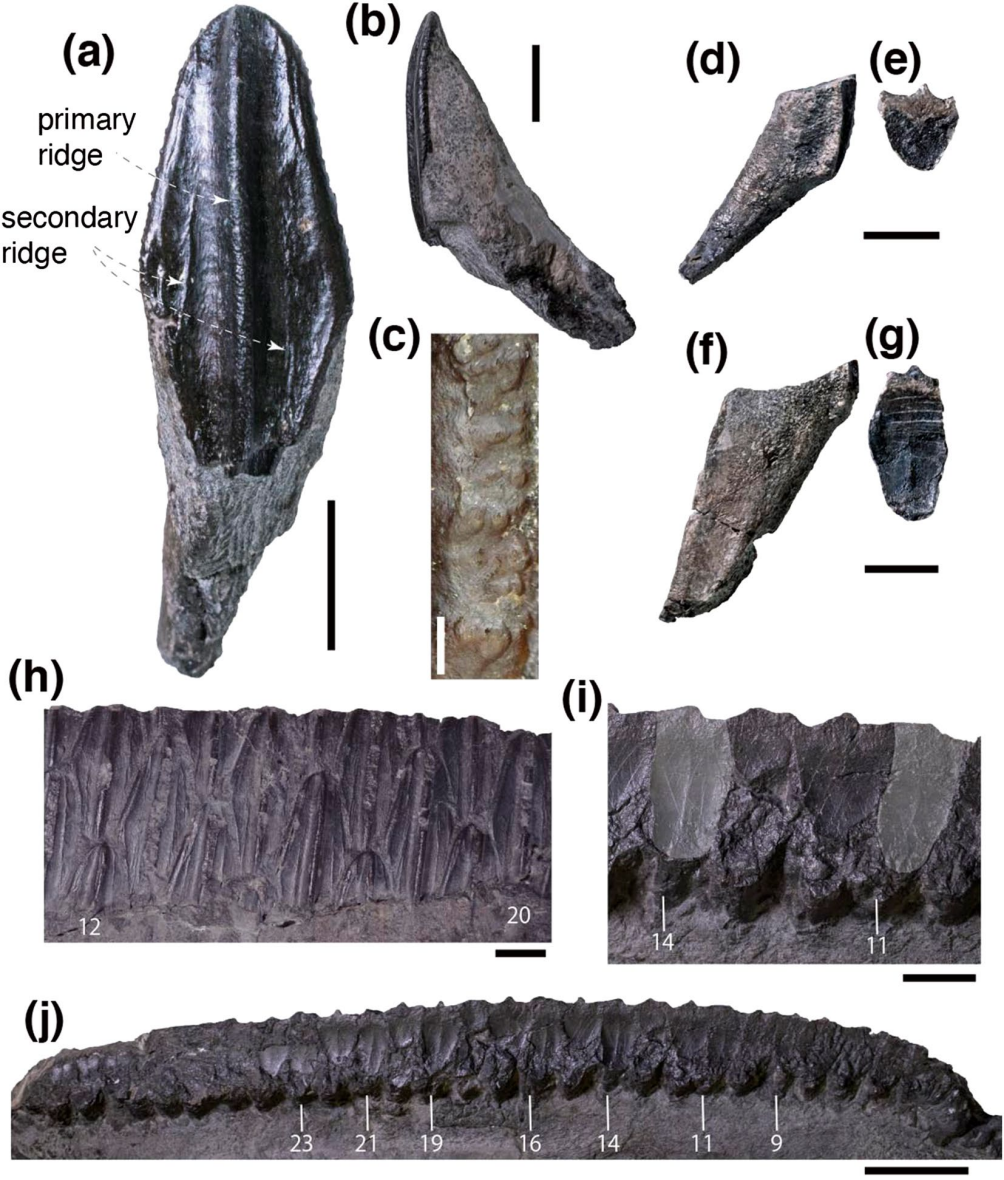
An isolated dentary tooth of *Yamatosaurus izanagii* gen. et sp. nov. from left side in lingual (**a**) and mesial (**b**) views and its denticles (**c**). Isolated dentary teeth of *Yamatosaurus izanagii* gen. et sp. nov. from left side in distal (**d**,**f**) and occlusal (**e**,**g**) views. Dentary teeth of *Yamatosaurus izanagii* gen. et sp. nov. in place on the right dentary in lingual (**h**) and occlusal (**i**) view. Numbers in (**h**), (**i**), and (**j**) are tooth positions. Occlusal surfaces of the eleventh and fourteenth teeth are highlighted in light gray in (**i**). Scales for (**a**), (**b**), and (**d**) to (**i**) are 1 cm. Scales for (**c**) and (**j**) are 0.5 mm and 3 cm, respectively.

At least 34 tooth positions are present, and each tooth position bears a maximum of four teeth in the right dentary, and at least twelve isolated dentary teeth from the left side are preserved. Tooth crowns are diamond-shaped and bear a prominent straight primary ridge in the middle teeth, while the primary ridge is sinuous in mesial and distal teeth (Figs. 2b, 4a,h). The secondary ridges are faint, wrinkled, and in some cases branched as in *Eotrachodon orientalis*^39^ and *Hypacrosaurus altispinus*^47^ but unlike the straight secondary ridges of *Corythosaurus casuarius* (ROM 868) and *Lambeosaurus lambei* (CMN 361, CMN 2869). The largest tooth crown, located in the middle portion of the dental battery, is approximately 41.94 mm high and 14.66 mm wide. The tooth crowns become shorter and narrower mesially and distally (Fig. 2b). The height/width ratios of the dentary tooth crowns are slightly less than three on average although vary largely by position. The marginal denticles along the mesial and distal edges of the coronal half of the tooth crown are proportionately smaller than those of *Eotrachodon orientalis*^39^ and *Lambeosaurus lambei* (CMN 351) and resemble those of *Hypacrosaurus altispinus* in size^47^. The marginal denticles of *Yamatosaurus izanagii* are formed of multiple small papillae (Fig. 4b,c), which are unorganized unlike in *Eotrachodon orientalis*^39^ and *Corythosaurus casuarius* (AMNH FARB 8527), but resembling *Gryposaurus notabilis* (AMNH FARB 8526). The angle between the crown and root of the dentary teeth is 132 degrees (Fig. 4b).

An occlusal surface of an isolated tooth changes from a flat pentagonal surface (Fig. 4d,e) to a concaved oval-shaped surface (Fig. 4f,g) as it wears. At most two teeth are functional per tooth position throughout the dental battery unlike hadrosaurids (e.g., *Edmontosaurus regalis*, CMN 2289; *Parasaurolophus tubicen*, NMMNH P-25100), which have more than two functional teeth in the middle of the dental battery. Only one functional tooth in 9th, 11th, 14th, 16th, 19th, 21st, and 23rd tooth positions (Fig. 4i,j) is unique to *Yamatosaurus izanagii*. The occlusal surface of the dental buttery is steeply inclined as in *Prosaurolophus maximus* (CMN 2277, CMN 2280), the distal area of the dental battery of *Brachylophosaurus canadensis* (CMN 8893), and the mesial area of the dental battery of *Gryposaurus* (*Gryposaurus notabilis*, ROM 873; *Gryposaurus latidens*, AMNH FARB 5465). The occlusal surface of *Yamatosaurus izanagii* bears low ridges that are associated with cementum and longitudinal giant tubules that fill the pulp cavity^13,48^. The occlusal topography is much lower than in *Corythosaurus*^13^ but resembles that of *Brachylophosaurus canadensis* (CMN 8893). The strong inclination and the low topography of the occlusal surfaces may suggest the pulp cavity of teeth of *Yamatosaurus izanagii* is filled mainly with transverse giant tubules, which are less wear-resistant than longitudinal giant tubules^13^.

**Figure 5:**
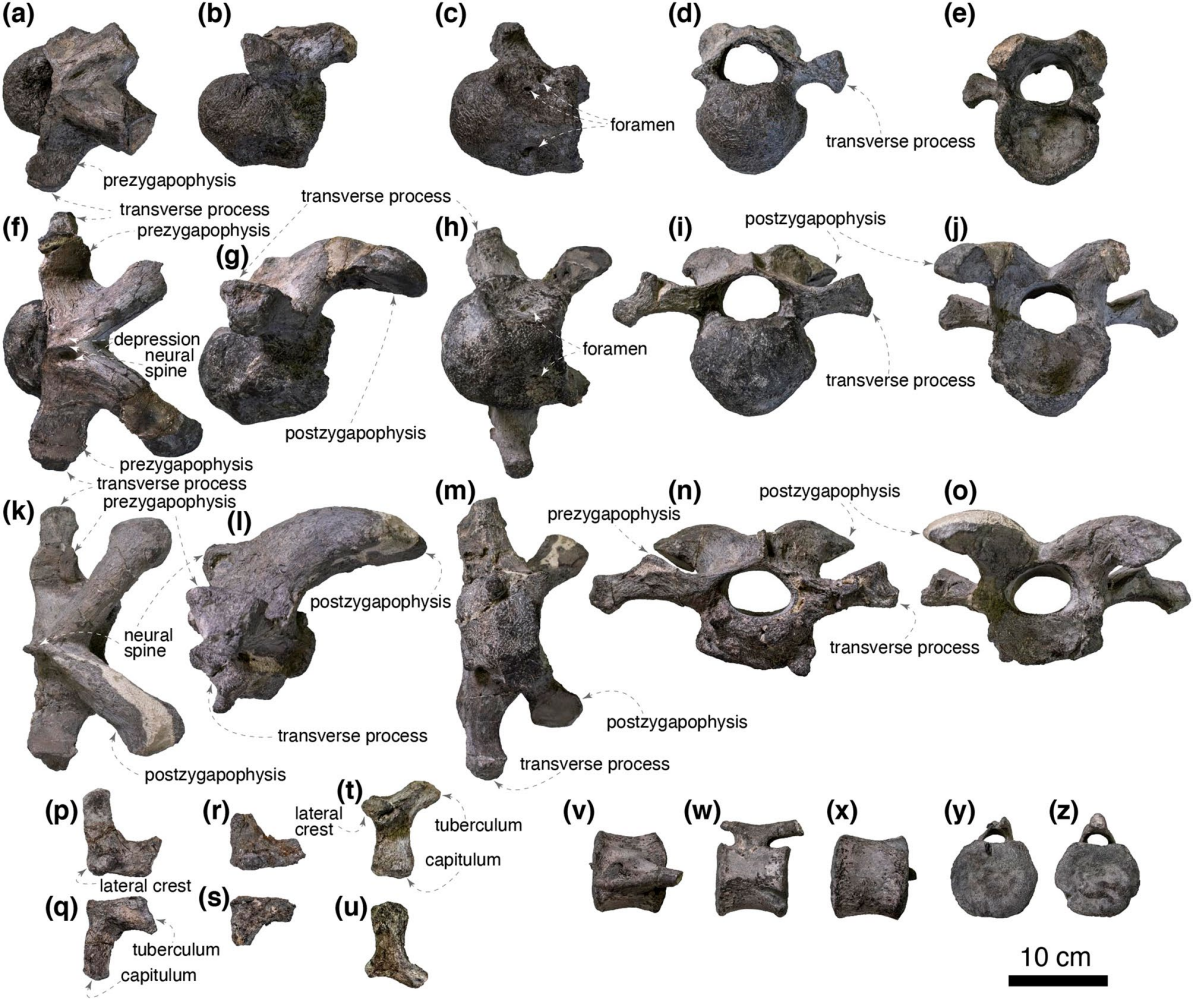
The fourth or fifth cervical vertebra of *Yamatosaurus izanagii* gen. et sp. nov. in dorsal (**a**), left lateral (**b**), ventral (**c**), anterior (**d**), and posterior (**e**) views. The fifth or sixth cervical vertebra of *Yamatosaurus izanagii* gen. et sp. nov. in dorsal (**f**), left lateral (**g**), ventral (**h**), anterior (**i**), and posterior (**j**) views. A middle cervical vertebra (seventh to ninth) of *Yamatosaurus izanagii* gen. et sp. nov. in dorsal (**k**), left lateral (**l**), ventral (**m**), anterior (**n**), and posterior (**o**) views. Cervical ribs of *Yamatosaurus izanagii* gen. et sp. nov. in dorsomedial (**p**,**r**,**t**) and ventrolateral (**q**,**s**,**u**) views. A distal caudal vertebra in dorsal (**v**), left lateral (**w**), ventral (**x**), anterior (**y**), and posterior (**z**) views.

At least four cervical vertebrae are preserved, and three of them are fairly complete (Fig. 5a–o). Although the exact position of these vertebrae is ambiguous, two are identified as anterior (fourth or fifth and fifth or sixth) and one as a middle (seventh to ninth) cervical vertebra based on comparisons with *Gobihadros mongoliensis* (MPC-D 100/746). The anterior cervical vertebrae are nearly complete other than postzygapophyses, whereas the middle cervical vertebra is missing the ventral half of its centrum. The anterior centra are strongly opisthocoelous. The anterior convexity and the posterior concavity are the most pronounced in the anteriormost cervical vertebra. The lateroventral surface of the centrum is deeply excavated and bears several foramina. The ventral surfaces of the centra are slightly convex and do not form a distinct keel. The neural canal is slightly wider than its height in the anteriormost cervical vertebra and becomes wider posteriorly. The transverse process is the shortest in the anteriormost cervical vertebra and becomes longer posteriorly. The dorsal surfaces of the prezygapophyses are slightly inclined mediodorsally. The diapophyses become longer posteriorly relative to the articular facets of the prezygapophyses. The neural spine is faint in the anteriormost cervical vertebrae and becomes larger posteriorly. Both sides of the base of the neural spine are shallowly depressed in the anteriormost cervical vertebra. The depressions are shallower and smaller in posterior cervicals. The postzygapophyses are less than three times as long as the anteroposterior length of the neural arch unlike hadrosaurids, including *Maiasaura peeblesorum* (ROM 44770) and *Hypacrosaurus stebingeri* (MOR 549) but resemble non-hadrosaurid hadrosauroids such as *Gilmoreosaurus mongoliensis* (AMNH FARB 6551). The postzygapophyseal articular surface is anteroposteriorly longer than mediolaterally wide in the anterior cervical vertebrae, whereas the articulation surface is subcircular in the middle cervical vertebra.

A distal caudal vertebra is nearly complete, missing a right prezygapophysis and a distal portion of the neural spine (Fig. 5v–z). The centrum is amphiplatyan and is hexagonal in anterior and posterior aspects. The lateral surfaces of the centrum bear a ridge in the middle, whereas the ventral surface is gently convex. The transverse process is absent as in the 38^th^ and more posterior caudal vertebrae of a lambeosaurine indet. (CMN 8330). The neural canal is a mediolaterally wide ellipsoid. The rod-shaped prezygapophysis lacks a distinct articular facet. The postzygapophysis is reduced and indistinguishable from the neural spine.

One right and two left cervical ribs are preserved (Fig. 5p–u). The capitulum is longer and more massive than the tuberculum in all preserved cervical ribs. The lateral crest is dorsoventrally flat and well-developed in the anterior-most preserved cervical rib and gradually diminishes posteriorly.

**Figure 6:**
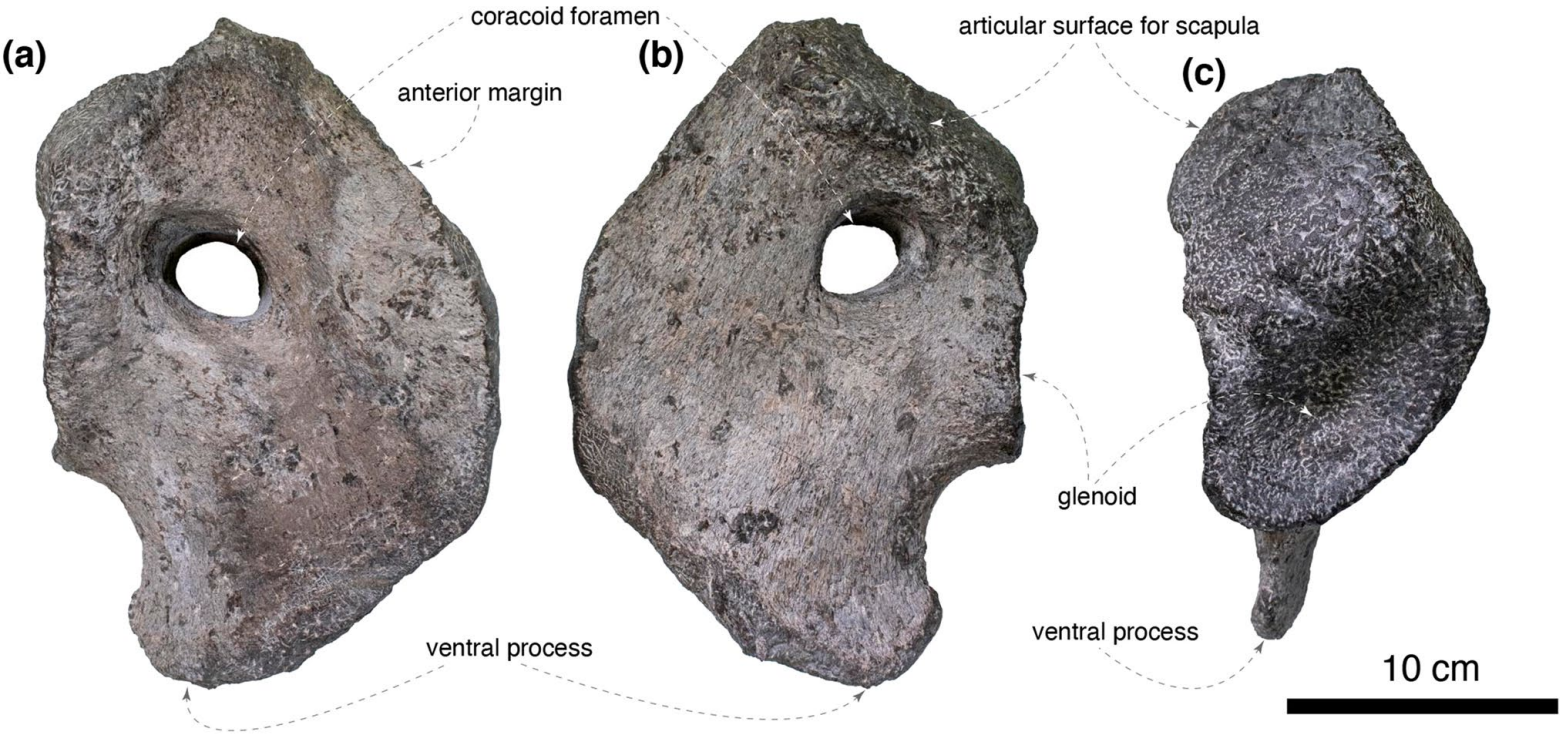
The right coracoid of *Yamatosaurus izanagii* gen. et sp. nov. in lateral (**a**), medial (**b**), and proximal (**c**) views.

The right coracoid is complete (Fig. 6). The articular facet for the scapula is shorter than the glenoid and angled 114° from the glenoid in the lateral view. The coracoid foramen is ellipsoid and does not intersect the scapulocoracoid contact. The anterior margin of the coracoid is straight. The biceps tubercle is absent. The ventral process is only half as long as its base as in non-hadrosaurid hadrosauroids (e.g., *Gilmoreosaurus mongoliensis*, AMNH FARB 30722), yet the ventral process is recurved caudoventrally as in hadrosaurids (e.g., *Brachylophosaurus canadensis*, MOR 1071 8-10-99-541; *Hypacrosaurus altispinus*, AMNH FARB 5272).

## Discussion

The ontogenetic stage of *Yamatosaurus izanagii* cannot be determined histologically due to the absence of any weight-bearing limb bones. However, the size of the preserved dentary (Supplementary Table S1) is comparable with those of large, presumably adult hadrosaurids including *Kamuysaurus japonicus* (526 mm)^19^ and *Hypacrosaurus stebingeri* (500 mm)^49^. In addition, the neurocentral sutures of preserved cervical vertebrae are closed (Fig. 5), suggesting the type specimen of *Yamatosaurus izanagii* was at least close to maturity at the time of its death^50,51^.

Our phylogenetic analysis produced 12 most parsimonious trees (MPTs) of 1181 steps, each with a consistency index of 0.430 and a retention index of 0.834. *Hadrosaurus foulkii*, which has been regarded as a *nomen dubium*^7,52^, is recovered as a sister taxon to the clade of *Yamatosaurus izanagii*, Saurolophinae, and Lambeosaurinae. Since the phylogenetic position of *Hadrosaurus foulkii* is comparable to that proposed by Prieto-Márquez^6^, this study follows that paper in the definitions of Hadrosauridae, Lambeosaurinae, and Saurolophinae.

**Figure 7:**
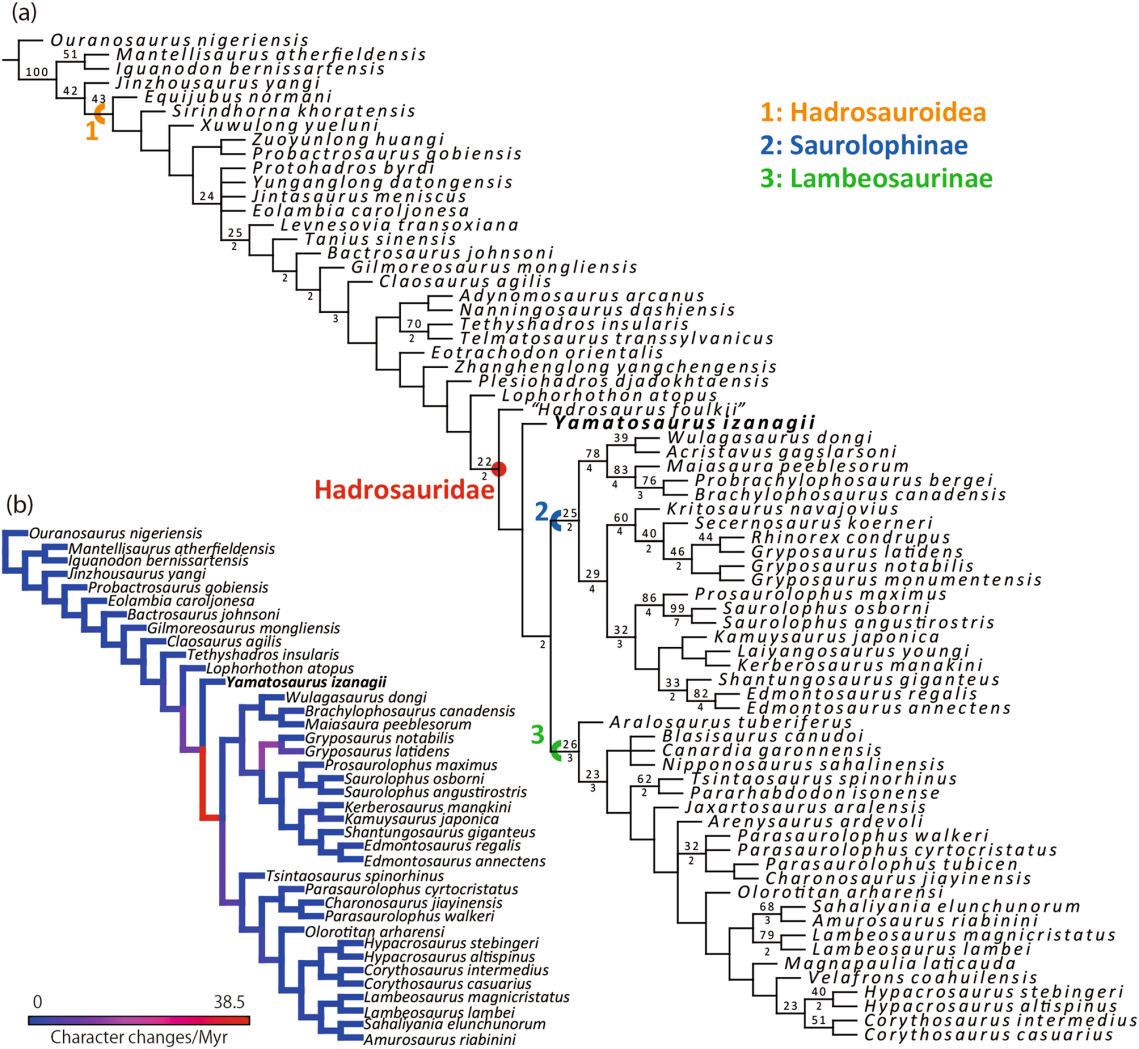
The strict consensus tree of the MPTs obtained from the phylogenetic analysis. The numbers above each branch represent bootstrap values and those below the branch represent the Bremer decay values. The bootstrap values below 20 and the Bremer decay values below 2 are not shown (**a**). Forelimb evolutionary rates on each branch based on the present phylogenetic hypothesis (**b**). Refer Supplementary Fig. S1 for the analysis without *Yamatosaurus izanagii*.

The cladistic analysis shows that *Yamatosaurus izanagii* belongs to Hadrosauridae, sharing two of five synapomorphies of the family (small denticles of dentary teeth and posteroventrally recurved ventral process of the coracoid), and is a sister taxon to the clade of Saurolophinae and Lambeosaurinae (Fig. 7). The clade of *Yamatosaurus izanagii*, Saurolophinae, and Lambeosaurinae is weakly supported, sharing a single synapomorphy (dentary tooth crown height-width ratio in between 2.70 and 3.30). *Yamatosaurus izanagii* is a sister taxon to the clade of Saurolophinae and Lambeosaurinae, which is well supported by five unambiguous characters: functional teeth at least two; symphysial region of the dentary weakly curved lingually; long postzygapophysis of the cervical vertebrae; concave craniodorsal margin of the coracoid; and presence of a well-developed biceps tubercle on the coracoid.

Three autapomorphies of *Yamatosaurus izanagii* are the crown and the root of the dentary teeth angled at between 110º and 130º, a low angle between the dentary symphysis and lateral surface of the dentary, and ventrally facing ventral surface of the surangular. The moderately angled dentary teeth crown and root is shared with derived lambeosaurines (Parasaurolophini + Lambeosaurini) and saurolophines other than Saurolophini and Kritosaurini. The low angle between the dentary symphysis and lateral surface of dentary is commonly seen in more primitive forms such as *Eotrachodon orientalis*, *Gilmoreosaurus mongoliensis*, *Bactrosaurus johnsoni*, and more primitive taxa, indicative of a reversal in *Yamatosaurus izanagii*. The ventrally facing surface of the surangular main body is a character for derived saurolophines (e.g., *Edmontosaurus* and *Saurolophus*) and lambeosaurines (e.g., *Corythosaurus* and *Hypacrosaurus*).

In addition to the autapomorphies recovered from the phylogenetic analysis, *Yamatosaurus izanagii* is unique among *Eolambia caroljonesa* and higher taxa in having only a single tooth as a minimum number of functional teeth per tooth position, even in the middle of the dentary dental battery (Fig. 4i,j). The dentary occlusal surface is also unique in the complete absence of the “branched ridges”^13^ in any of the preserved teeth. Since the ridges are formed at the plugged pulp cavity^48,53^, the absence of the “branched ridges” may indicate a unique tooth ontogeny of *Yamatosaurus izanagii* although histological observation is mandatory to test tooth ontogeny. Other than the dentition, the *Yamatosaurus izanagii* dentary exhibits derived characters. It differs from the other derived non-hadrosaurid hadrosauroids, including *Eotrachodon orientalis*^39^, *Zhanghenglong yangchengensis*^7^, and *Plesiohadros djadokhtaensis*^41^, in having the ventral deflection of the anterior dentary, the posterior elongation of the dental battery, and the anteromedial elongation of the symphysial process.

While the dentary possesses multiple derived features, the coracoid of *Yamatosaurus izanagii* resembles those of non-hadrosaurid hadrosauroids such as *Eolambia caroljonesa* and *Gilmoreosaurus mongoliensis* in its straight anterior margin, short ventral hook, and undeveloped biceps tubercle. Dilkes^54^ interpreted the biceps tubercle of hadrosaurids as the origin of *m. biceps*, which extends to the proximal ends of the ulna and radius. Since *m. biceps* functions to lift the antebrachium, the presence and enlargement of the biceps tubercles in saurolophines and lambeosaurines may represent a functional change in forelimb use. The evolutionary rate analyses on pectoral girdle + forelimb phylogenetic characters demonstrate a significantly high evolutionary rate in the branch leading to the clade of Saurolophinae and Lambeosaurinae (Fig. 7b), as suggested by Stubbs et al.^55^ in the postcranial characters. Although one concern of this high evolutionary rate is that it could be an artifact of the incompleteness of *Yamatosaurus izanagii,* as warned by Lloyd^56^, but the same trend is recovered in an analysis without *Yamatosaurus izanagii* (Supplementary Fig. S1) strengthening our supposition. Therefore, the pectoral girdle + forelimb morphology is likely to have experienced rapid morphological changes at the base of Hadrosauridae. The rapid pectoral girdle + forelimb modifications may be associated with hadrosauroid gait change. Although Maidment and Barrett^57^ suggested that facultative quadrupedality was acquired more basal to *Gilmoreosaurus mongoliensis*, the evolutionary rates of the pectoral girdle + forelimb may suggest the gait shift was accomplished in basal hadrosaurids. Previously, changes in jaw elements and dentition have been considered as key features for the origin of Hadrosauridae, but this study adds that forelimb locomotion, resulting from shoulder and forelimb structural modification, is also an important feature within the origin of this family.

**Figure 8:**
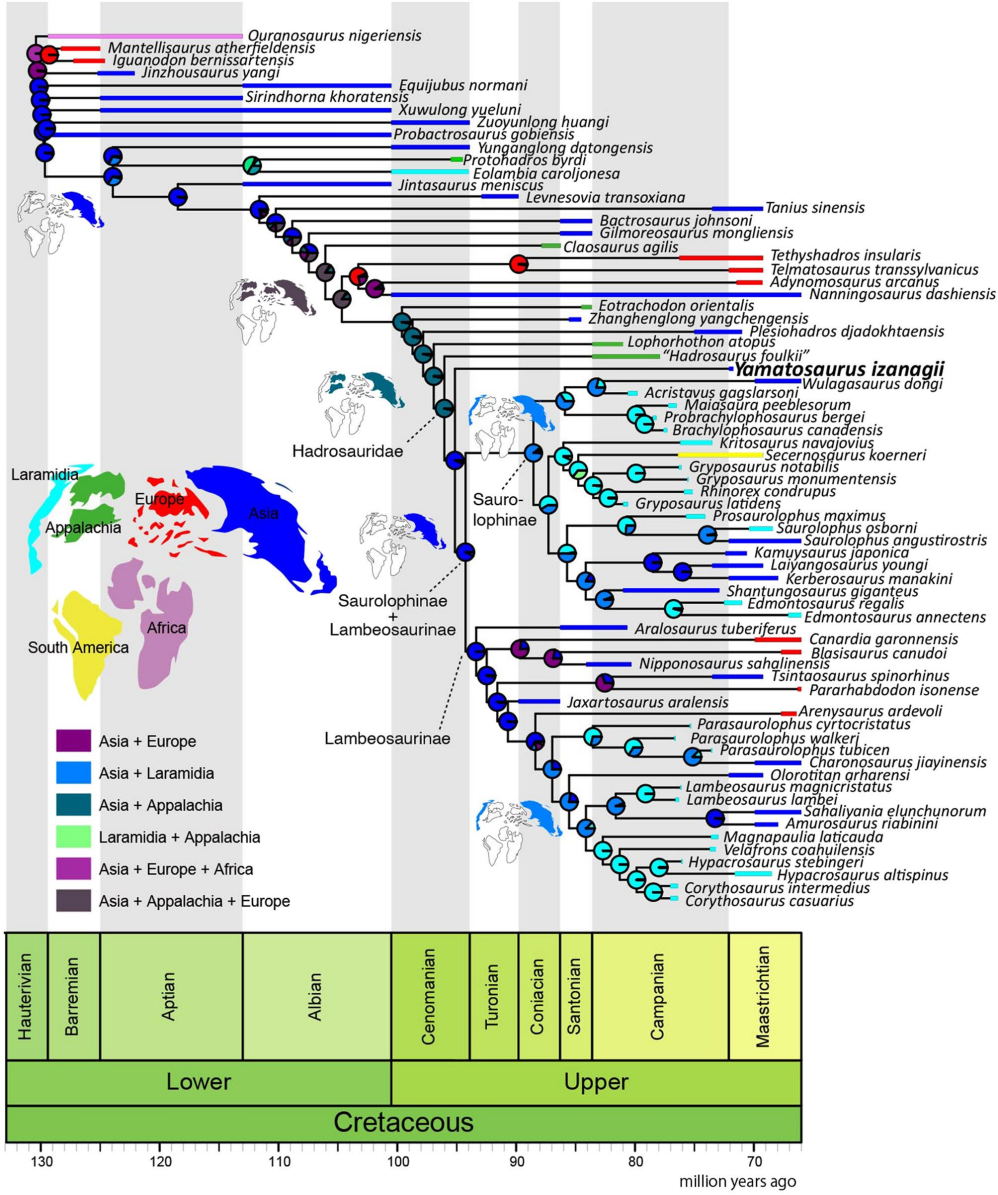
Result of the biogeographic analysis using the “starting” dispersal multiplier matrix. Refer Supplementary Figs. S1, S2 for the analyses using the “relaxed” and “harsh” dispersal multiplier matrix.

The biogeographic analyses resulted in higher fits in the analyses using the “starting” and the “relaxed” dispersal multiplier matrices than in the “harsh” (Supplementary Table S2). The results suggest that permitting intercontinental dispersal probabilities can explain hadrosaurid dispersal history better than considering that intercontinental dispersal was nearly impossible. The results in the “starting” and the “relaxed” matrices are nearly identical to each other, thus only the “starting” result is presented here (Fig. 8; see Supplementary Figs. S2 and S3 for the “relaxed” and “harsh” results). The analysis suggested that successive nodes of derived hadrosauroids from *Eotrachodon orientalis* to the node of Hadrosauridae were widely distributed in Asia and Appalachia, but not the intervening landmasses of Laramidia or Europe. Previous studies argued that the largely diversified clade of Saurolophinae and Lambeosaurinae (Hadrosauridae sensu Xing et al.^7^) originated in North America^11,58^ or Asia^5^, but our analysis supports the Asian origin of this clade thus refining other previous broader models of ornithopod origins in Asia^59^ while also contributing to a better understanding of the origins of high latitude dinosaurs^60^. Further biogeographic resolution will be aided with additional specimens, especially from pre-Campanian Upper Cretaceous deposits, because the successive taxa of non-hadrosaurid hadrosauroids, especially from *Eotrachodon orientalis* to *Plesiohadros djadokhtaensis*, have long ghost lineages up to 20 my. *Yamatosaurus izanagii* shows the longest ghost lineage duration of roughly 30 my among hadrosaurids. Interestingly, given the length of the ghost lineages of the Maastrichtian non-hadrosaurid hadrosauroids *Plesiohadros djadokhtaensis* from Mongolia and *Tanius sinensis* from China, considered with the ghost lineage represented by *Yamatosaurus izanagii*, it may be that eastern Asia (Japan, Mongolia and China) served as a refugium of relict hadrosauroid taxa.

Hadrosauroid materials have been reported from the Upper Cretaceous deposits of Japan^20^, and the youngest occurrence is *Kamuysaurus japonicus* from the early Maastrichtian marine deposits of the Hakobuchi Formation in Mukawa Town in Hokkaido Prefecture^19^, which is equivalent age to *Yamatosaurus izanagii*. Although the paleo-latitudes of Mukawa Town and Sumoto City during the Cretaceous are estimated to be similar to the current position (approximately 1100 km apart), it is clear that these dinosaurs were present at the same geologic time. It was suggested that the non-hadrosaurid hadrosauroid *Plesiohadros djadokhtaensis* in late Campanian was replaced by hadrosaurids such as *Saurolophus angustirostris* and *Barsboldia sicinskii* in Maastrichtian in Mongolia, indicating that the co-existence of hadrosaurid and non-hadrosaurid hadrosauroid is unlikely in the fluvial depositional environments^41^. The replacement of hadrosauroids by more derived members during the Maastrichtian is suggested in Laurasia^41^ but not in Europe (*Telmatosaurus transsylvanicus* and *Tethyshadros insularis*) because the European archipelago persisted as island relicts^3,61,62^. The occurrence of *Yamatosaurus izanagii* and *Kamuysaurus japonicus* from the time equivalent formations shows the contemporaneous existence of a derived and a basal hadrosaurid in Asia for the first time (Fig. 1g). This contemporaneous occurrence may be due to a different paleoenvironment preserved where Japan was more coastal in nature and richer in vegetation with plants such as plane trees (*Platanus* sp.), elm (*Ulmus* sp.), and cycads (*Zamiophyllum* sp.) than the fluvial environments preserved in the mainland Asian continent. An alternative scenario is that these dinosaurs did not co-occur and instead there was some level of provinciality within the coastal environments of this part of Asia where *Kamuysaurus japonicus* occupied a northern area and *Yamatosaurus izanagii* was restricted to a more southern area.

## Materials and methods

### Phylogeny

Phylogenetic analysis was conducted on the data matrix modified from Takasaki et al.^63^, using TNT ver 1.5^64^. The immature *Edmontosaurus* OTUs were removed from the original data matrix since their phylogenetic relationships are out of the scope of this study. *Nanyangosaurus zhugeii* was also excluded from the analysis because of its poor preservation. *Protohadros byrdi* is included in the data matrix by scoring the phylogenetic characters based on the published literature^65^. *Lophorhothon atopus* was re-scored based on the recently published paper by Gates and Lamb^66^. The resultant data matrix consists of 71 OTUs and 354 characters (Supplementary Data S1). A phylogenetic analysis was conducted using TNT ver. 1.5^64^, with *Ouranosaurus nigeriensis* as the outgroup. All of the characters were equally weighted and unordered. The maximum number of trees was set to 99,999 in memory. A traditional search with 10,000 replicates of Wagner trees using random additional sequences, followed by the TBR branch swapping that held 10 trees per replicate was performed. Supports for the clade were evaluated by bootstrap resampling using standard absolute frequencies (1000 replicates) and Bremer decay indices calculations.

### Evolutionary rates

Branch evolutionary rates of the phylogenetic characters related to pectoral girdle + forelimb were examined using R package Claddis version 0.6.5^56^ in R v.4.0.3^67^, based on the current morphological data matrix and the phylogenetic hypothesis. OTUs with character completeness less than 50% are pruned prior to the analyses since missing data leads to extreme results^56^. Removal of the taxa with low character completeness also provides much better resolution to the phylogenetic tree. The remaining polytomies were randomly resolved prior to the branch partition analyses then given branch lengths of 10^–6^ of the total tree lengths. One-hundred time-calibrated phylogenetic trees which incorporate the ranges of the time of first and last appearances (Supplementary Data S2) were constructed based on the equal method^68^ using the R package paleotree^69^. The analysis was repeated twice, with and without *Yamatosaurus izanagii*. The analysis was repeated twice, with and without *Yamatosaurus izanagii*. The analyses are conducted using the R script provided as the Supplementary Data S3.

### Ancestral range reconstruction

Ancestral habitat ranges were reconstructed using R package BioGeoBEARS^70^ in R v.4.0.3^67^ using the R script in the Supplementary Data S4. Although recent biogeographic works generally conduct analyses over multiple models^58,71^, Ree and Sanmartín^72^ recently pointed out conceptual problems in “+J” models. Since our interest is in habitat range reconstruction rather than model selection, this study conservatively performed only the DEC analysis following the suggestions by Ree and Sanmartín^72^. A phylogenetic hypothesis based on the majority consensus tree was used. The remaining polytomies were resolved then given branch lengths of 10^–6^ of the total tree lengths. To incorporate the ambiguities in time ranges of the OTUs, 100 time-calibrated phylogenetic trees were produced using the R package paleotree^69^, based on the “equal” method^68^. The marginal probabilities at each node are averaged over the 100 time-calibrated trees. Three analyses with the “starting”, “harsh”, and “relaxed” dispersal multiplier matrices were conducted following Poropat et al.^73^. In the “starting” and the “relaxed” matrices, the dispersal multiplier values between the following continents are set to 0.5 and 1, respectively: Asia and Laurasia, Asia and Europe, Laramidia and Appalachia, Laramidia and South America, Appalachia and Europe, and Europe and Africa (Supplementary Data S5–S7). The maximum range size allowed for any species to occupy was set to 3. The dates and the continents of each OTUs are compiled from previous literature (Supplementary Data S2, S8).

## Supplementary Information

Supplementary Information 1.

Supplementary Information 2.

Supplementary Information 3.

Supplementary Information 4.

Supplementary Information 5.

Supplementary Information 6.

Supplementary Information 7.

Supplementary Information 8.

Supplementary Information 9.

Supplementary Information 10.
