# Supplementary material for: A new basal hadrosaurid (Dinosauria: Ornithischia) from the latest Cretaceous Kita-ama Formation in Japan implies the origin of hadrosaurids
Source: Sci Rep. 2021 Apr 27;11:8547. doi: 10.1038/s41598-021-87719-5 (PMC8076177; doi:10.1038/s41598-021-87719-5)
Supplement: Supplementary file 1 — Supplementary Information 1. [file 41598_2021_87719_MOESM1_ESM.pdf]

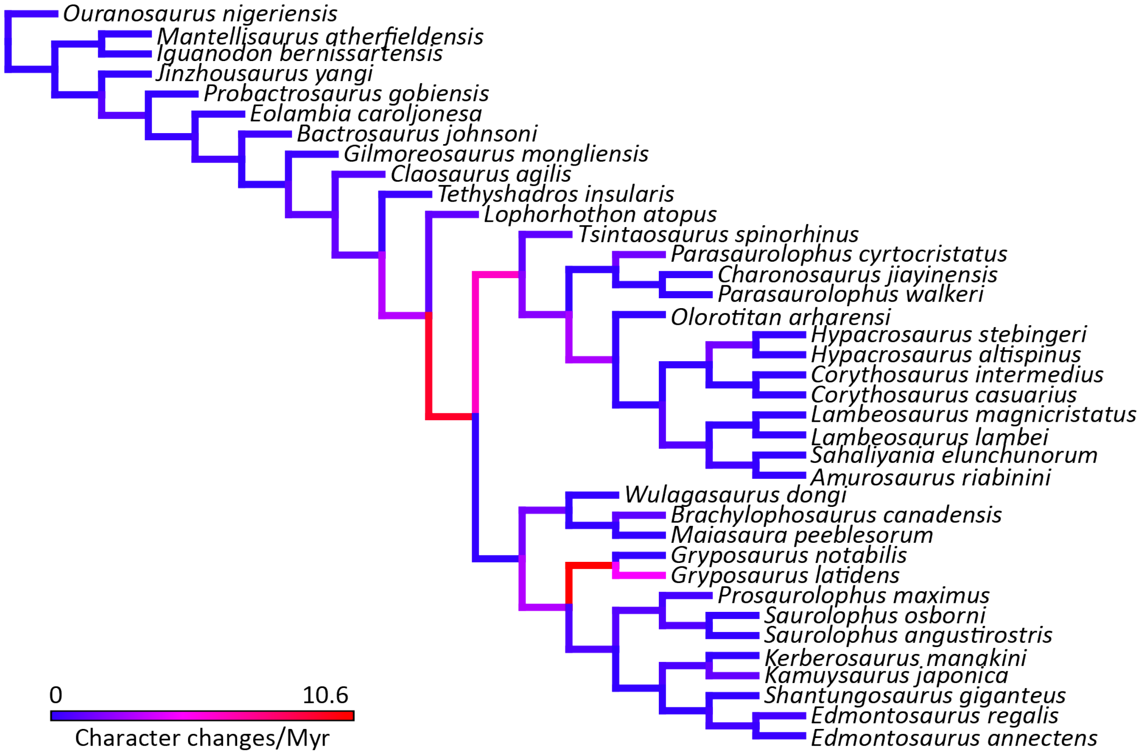

Supplementary Figure S1. Forelimb evolutionary rates on each branch based on the present phylogenetic hypothesis without *Yamatosaurus izanagii*.

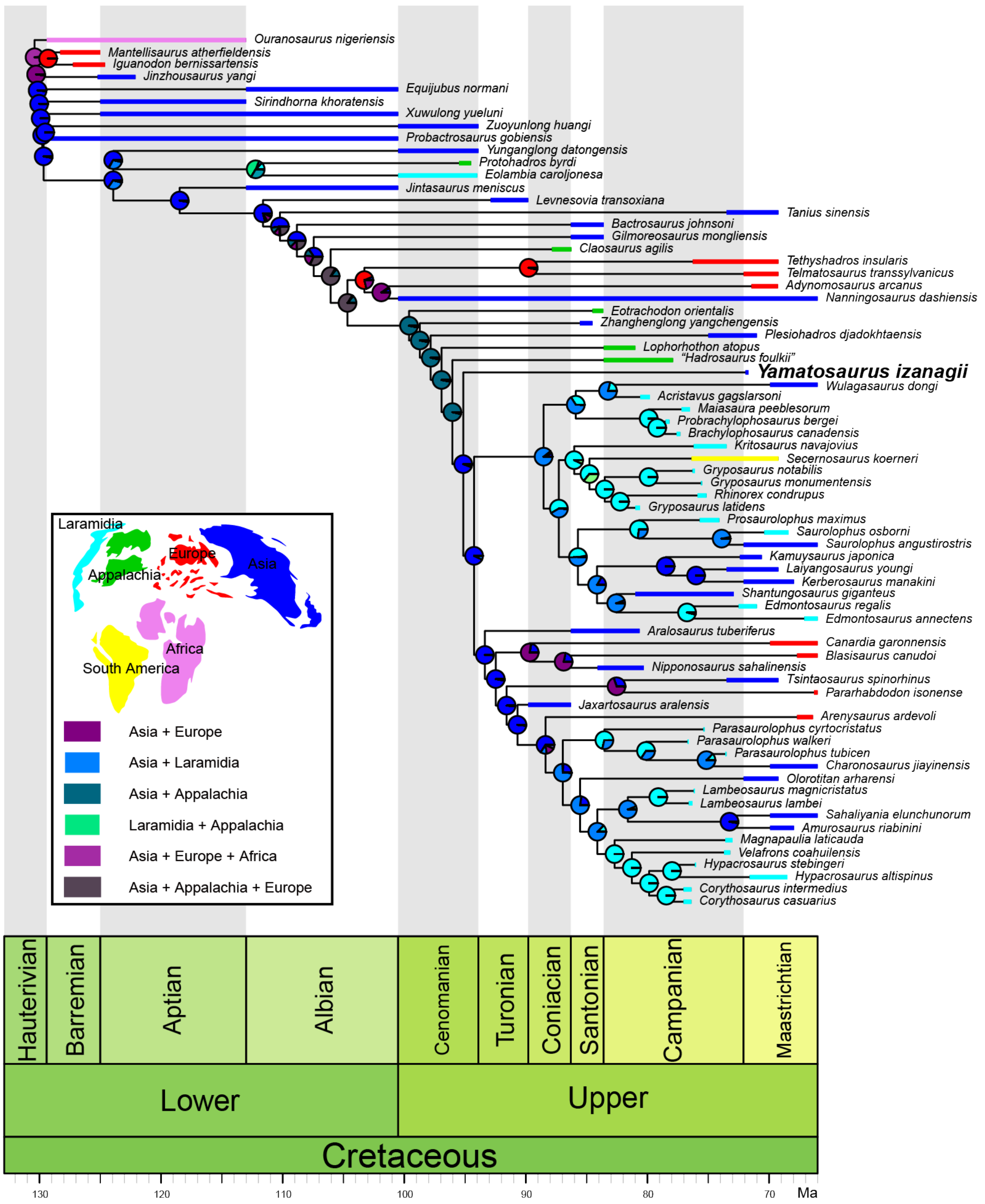

Supplementary Figure S2. Result of the biogeographic analysis using the "relaxed" dispersal multiplier matrix.

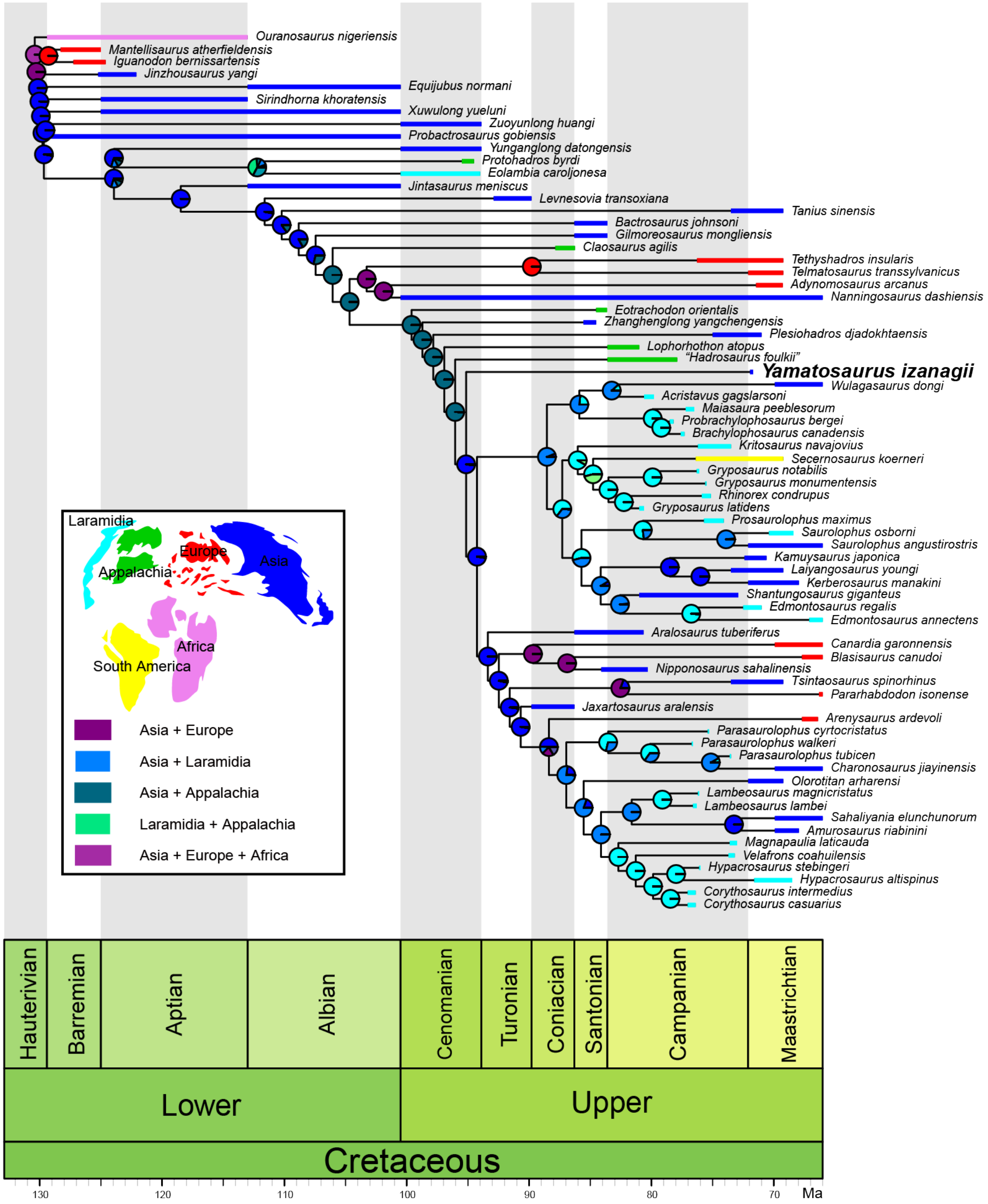

Supplementary Figure S3. Result of the biogeographic analysis using the "harsh" dispersal multiplier matrix.
